# Persistently Active Microbial Molecules Prolong Innate Immune Tolerance In Vivo

**DOI:** 10.1371/journal.ppat.1003339

**Published:** 2013-05-09

**Authors:** Mingfang Lu, Alan W. Varley, Robert S. Munford

**Affiliations:** 1 Laboratory of Clinical Infectious Diseases, National Institute of Allergy and Infectious Diseases, National Institutes of Health, Bethesda, Maryland, United States of America; 2 Infectious Diseases Division, Department of Internal Medicine, University of Texas Southwestern Medical Center, Dallas, Texas, United States of America; University of São Paulo, Brazil

## Abstract

Measures that bolster the resolution phase of infectious diseases may offer new opportunities for improving outcome. Here we show that inactivation of microbial lipopolysaccharides (LPS) can be required for animals to recover from the innate immune tolerance that follows exposure to Gram-negative bacteria. When wildtype mice are exposed to small parenteral doses of LPS or Gram-negative bacteria, their macrophages become reprogrammed (tolerant) for a few days before they resume normal function. Mice that are unable to inactivate LPS, in contrast, remain tolerant for several months; during this time they respond sluggishly to Gram-negative bacterial challenge, with high mortality. We show here that prolonged macrophage reprogramming is maintained *in vivo* by the persistence of stimulatory LPS molecules within the cells' *in vivo* environment, where naïve cells can acquire LPS via cell-cell contact or from the extracellular fluid. The findings provide strong evidence that inactivation of a stimulatory microbial molecule can be required for animals to regain immune homeostasis following parenteral exposure to bacteria. Measures that disable microbial molecules might enhance resolution of tissue inflammation and help restore innate defenses in individuals recovering from many different infectious diseases.

## Author Summary

We showed previously that mice lacking acyloxyacyl hydrolase (AOAH), the host enzyme that inactivates Gram-negative bacterial lipopolysaccharides (LPS), are unable to regain normal immune responsiveness for many weeks/months after they are exposed *in vivo* to a small amount of LPS or Gram-negative bacteria. The many possible explanations for slow recovery included long-lasting epigenetic changes in macrophages or other host cells, chronically stimulated cells that produce certain mediators, and persistent signaling by internalized LPS within macrophages. Using several *in vivo* techniques to study peritoneal macrophages, we found that none of these mechanisms was correct. Rather, prolonged recovery is caused by intact LPS that remains in the environment where macrophages live and can pass from one cell to another *in vivo*. This is the first evidence that the persistence of a bioactive microbial agonist, per se, can prevent resolution of inflammation *in vivo*. It also identifies the stimulatory microbial molecule as a realistic target for intervention – in further support, we found that providing recombinant AOAH can be partially preventive. In a larger sense, showing that chemical inactivation of one important microbial signaling molecule is required for full recovery should encourage efforts to find out whether disabling other microbial agonists (chitin, lipopeptides, flagella, others) also benefits infected animals.

## Introduction

A great deal is known about how animals kill bacteria but very little about what happens to the corpses. Most bacterial structures seem to be broken down by phagocytes [1], [2], yet much of the catabolic machinery is made by the bacteria themselves (phospholipases [3], peptidoglycan hydrolases [4], autolysins), which in some cases produce agonists that can elicit inflammation. Host enzymes may also participate, attacking peptidoglycan (lysozyme, peptidoglycan binding proteins [5], [6]), lipids (phospholipases [3]), chitin [7], proteins (cathepsins, others), lipopolysaccharides (LPS) and, presumably, DNA. It is likely that the breakdown products are excreted, retained within phagocytes (e.g., in lymph nodes or arterial walls [8], [9]) or in discrete extracellular deposits [10], or recycled by the host.

The seminal studies of Cohn [1], [11], Elsbach [2], [3] and Schwab [12] established that degradation of ingested bacteria by phagocytes can be incomplete. More recently, deposits of microbial antigens have been discovered in animals following infection with *B. burgdorferii*, the etiological agent of Lyme Disease [10]. A key question raised by these findings has remained unanswered to date: to what extent does the inactivation of stimulatory microbial molecules influence the outcome of infectious diseases or host reactions to microbe-rich environmental exposures? Here we show that for one important agonist, bacterial lipopolysaccharide (LPS), persistently active molecules can greatly delay immune recovery *in vivo*.

Exposure to Gram-negative bacterial LPS induces humans and many other mammals to enter a transient state of altered immune responsiveness known as cellular reprogramming or tolerance [13]–[15]. The phenomenon has also been produced in animals by peritonitis [16], influenza virus infection [17] and bacterial lipopeptides [18], [19] and in cultured macrophages using muramyl dipeptide [20], lipoteichoic acid [21], and flagella [22]. For a period that may last from a few hours to a few days after exposure to the microbial agonist, tolerant animals and cells respond to a second exposure by producing reduced amounts of many pro-inflammatory cytokines while maintaining, or even increasing, their production of certain anti-inflammatory and anti-infective molecules. Tolerance wanes as inflammation resolves and the animal regains normal immune responsiveness. Long considered a mechanism for preventing inflammation-induced injury, innate immune tolerance may also be immunosuppressive [23].

Acyloxyacyl hydrolase (AOAH), an enzyme produced by macrophages, neutrophils, and dendritic cells, inactivates bioactive LPSs by removing two of the six fatty acyl chains that are present in the bioactive hexaacyl lipid A moiety [24]. In mice that lack AOAH, a single intraperitoneal exposure to as little as 80 ng of hexaacyl LPS induces tolerance that lasts for several weeks, much longer than that seen in wildtype animals that have received much larger doses [25]. Tolerant animals respond sluggishly to Gram-negative bacterial challenge and are unable to prevent bacterial multiplication *in vivo* [25].

The mechanism(s) by which fully acylated LPS maintains reprogramming *in vivo* have been uncertain. Here we considered several possibilities. First, it seemed likely that bioactive LPS would remain within or on *Aoah^−/−^* macrophages for prolonged periods and render them tolerant via cell-intrinsic signaling, despite the existence of mechanisms that promote LPS efflux from macrophages [26], [27]. Second, it was possible that extracellular bioactive LPS, released from tolerant macrophages and/or other *in vivo* reservoirs, could prevent tolerant macrophages from recovering and induce tolerance in recruited naïve monocytes. A third consideration was that mediators produced by tolerant cells, even in the absence of LPS, could induce tolerance in themselves and other cells *in vi*vo [21]. Finally, it was possible that LPS-stimulated *Aoah^−/−^* macrophages might undergo long-lived, stable reprogramming that persisted even in the absence of bioactive LPS [28]–[32]. A combination of these mechanisms was also considered.

The studies described here indicate that macrophage tolerance can be maintained for long periods *in vivo* by the presence of small amounts of fully acylated extracellular LPS. The source of the LPS seems to be extrinsic to the tolerant cell, coming from the fluid in which macrophages live or the LPS-containing cells they contact *in vivo*. We found that cell-associated LPS can be released, bind to other cells, and induce or maintain their tolerant state. Importantly, LPS-exposed, tolerant macrophages regained normal responsiveness when transferred to a LPS-free environment. Furthermore, *in vivo* inactivation of LPS by administering recombinant AOAH partially prevented tolerance. These results identify persistence of bioactive LPS in both cells and cell-extrinsic reservoirs as a primary mechanism that drives prolonged macrophage tolerance *in vivo.* They suggest that measures to inactivate LPS in these reservoirs might shorten the period of macrophage unresponsiveness that follows many Gram-negative bacterial diseases. They also provide evidence that inactivation of microbial molecules can be an essential element of the resolution/recovery phase of infectious illnesses.

## Results

### LPS exposed, tolerant *Aoah^−/−^* macrophages have distinctive features

**Figure 1:**
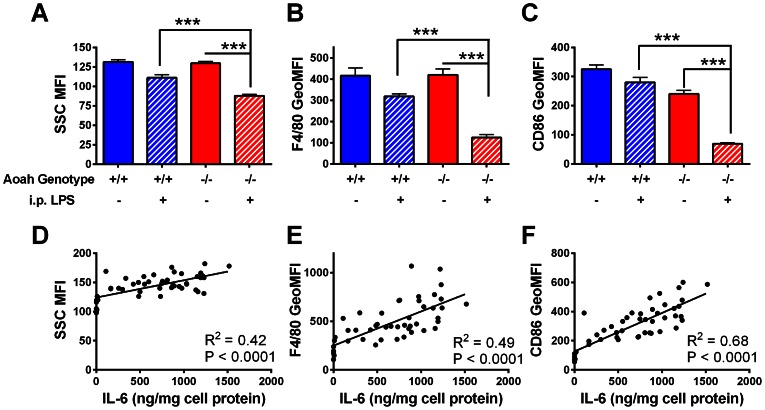
Tolerant peritoneal macrophages from LPS-injected *Aoah^−/−^* mice have distinctive characteristics. (**A–C**) *Aoah^+/+^* or *Aoah^−/−^* mice were injected i.p. with PBS or 10 µg *E. coli* O14 LPS. Fourteen days later, their peritoneal cells were harvested; the F4/80+ cells (macrophages) were identified using flow cytometry and their SSC (**A**), surface expression of F4/80 (**B**) and CD86 (**C**) were measured. Data shown are from 2 independent experiments. n = 6–8. ***, p<0.001. When compared with the other groups, LPS-exposed *Aoah^−/−^* macrophages have lower SSC and express less surface F4/80 and CD86. Blue bars, *Aoah^+/+^*; Red bars, *Aoah^−/−^*; diagonal markings indicate i.p. LPS injection. (**D–F**) **Macrophage IL-6 production following LPS re-stimulation correlates with surface marker expression.** *Aoah^+/+^* or *Aoah^−/−^* mice were injected with 0, 0.016, 0.08, 0.4, 2 or 10 µg *E. coli* O14 LPS i.p. Each LPS dose was given to 3–5 *Aoah* ^+/+^ and *Aoah^−/−^* mice. Fourteen days later, their peritoneal cells were removed. Some were used to measure surface markers F4/80, CD86 and SSC by flow cytometry. Others were cultured for 18 hours, the floating cells were washed away and the adherent macrophages were re-stimulated with *E. coli* O111 LPS (1 µg/ml) for 6 hours. Medium IL-6 levels were measured by ELISA and correlated with SSC, F4/80 and CD86 expression on macrophages from the same mice. Each dot represents data from one mouse. Secreted TNF concentrations also positively correlated with these markers (**Fig. S1**). MFI; mean fluorescence intensity. Geo MFI; geometric mean fluorescence intensity.

As previously reported [25], 14 days after LPS injection, *Aoah^+/+^* mice have largely recovered from tolerance while *Aoah^−/−^* mice are still reprogrammed. To identify phenotypic markers of tolerance, we injected *Aoah^−/−^* and *Aoah^+/+^* mice i.p. with LPS or PBS and isolated their peritoneal macrophages 14 days later. Peritoneal macrophages (F4/80+ cells as determined by flow cytometry) from LPS-injected *Aoah^−/−^* mice had lower SSC (granularity), less surface F4/80 (macrophage marker, EGF-TM7 member of the adhesion-GPCR family), and less surface CD86 (costimulatory molecule, also reduced during innate immune tolerance in humans [33]) when compared with their LPS-exposed *Aoah^+/+^* counterparts (**Fig. 1 A–C**). Each of these parameters correlated with the cells' responses to LPS *ex vivo* (**Fig. 1 D–F** **, Fig. S1**). When compared with naïve *Aoah^−/−^* or LPS-exposed *Aoah^+/+^* macrophages, macrophages from LPS-exposed *Aoah^−/−^* mice also had less surface CD11b (macrophage marker, Integrin αM chain), CD69 (early activation marker), CD16/32, CD32 (Fcγ receptors), and Ly6C/G (Gr1) and slightly higher CD40 (costimulatory molecule) expression (**Table S1**). They did not have higher expression of two markers found in alternatively activated macrophages, arginase or FIZZ 1 (**Table S1**). Based on these findings, we used SSC and surface F4/80 and CD86 expression to identify the tolerant phenotype in subsequent experiments.

### Macrophages retain intracellular LPS for long periods *in vivo*

**Figure 2:**
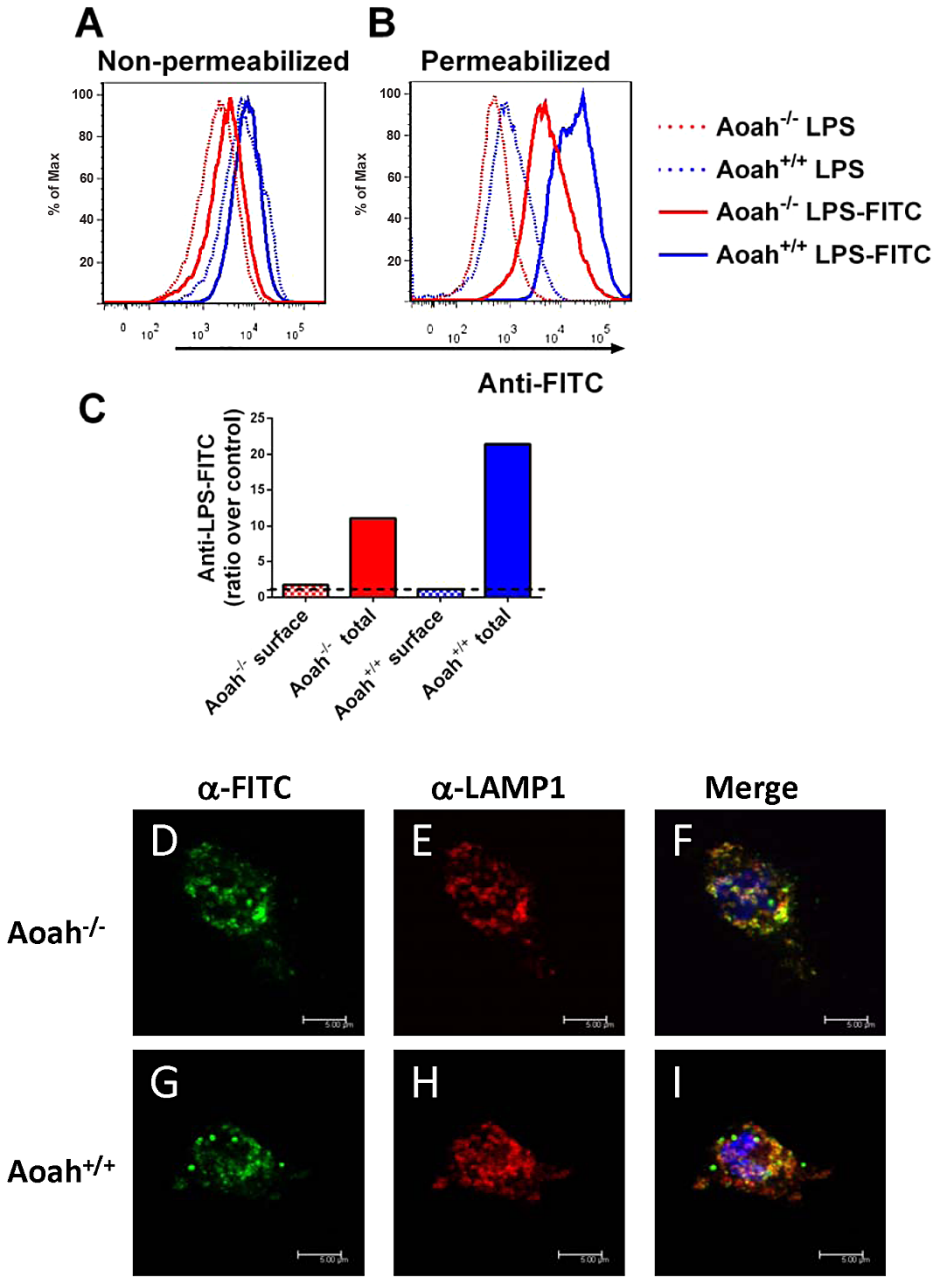
Cell-associated LPS. *Aoah^+/+^* or *Aoah^−/−^* mice were given 10 µg LPS-FITC i.p. Ten days later, their peritoneal cells were harvested, fixed, and either permeabilized (**B**) or not (**A**). Anti-FITC Ab conjugated with Phycoerythrin (PE) was added to measure macrophage (F4/80+) surface (**A**) or total (**B**) LPS-FITC. Dotted lines, F4/80+ cells from mice that received LPS i.p. (control for autofluorescence); solid lines, F4/80+ cells from mice that were given i.p. LPS-FITC. Red lines, *Aoah^−/−^* ; blue lines, *Aoah^+/+^*. (**C**) The ratio of the GeoMFI of cell surface or total LPS-FITC to the GeoMFI of control macrophages. The dotted line indicates a ratio of 1.0 (no LPS-FITC association with cells). For both *Aoah^−/−^* and *Aoah^+/+^* cells, most of the FITC was intracellular. (**D–I**) Mice were given 10 µg LPS-FITC i.p. Ten days after injection, peritoneal cells were harvested and cultured in plates with coverslips for 18 hours. Floating cells were washed away and adherent macrophages were fixed, permeabilized, and stained with anti-FITC Alexa fluor 488 (green), anti-LAMP1 (CD107) Ab (red) and DAPI (blue). (**D and E**) *Aoah^−/−^* macrophages. **F**, overlay of **D** and **E**. (**G and H**) *Aoah^+/+^* macrophages. **I**, overlay of **G** and **H**. Much of the FITC-LPS was localized to LAMP1-positive vesicles in both *Aoah^−/−^* and *Aoah^+/+^* macrophages. Scale bar = 5 µm.

We previously found that both *Aoah^+/+^* and *Aoah^−/−^* peritoneal macrophages retain LPS for at least 10 days *in vivo* [25]. Whereas the LPS in *Aoah^+/+^* macrophages had been partially deacylated (i.e., it had lost two of the six fatty acyl chains from lipid A), that in *Aoah^−/−^* macrophages was fully acylated [25]. The cells' ability to produce TNF in response to a second exposure to LPS was related inversely to their LPS content; *Aoah^+/+^* macrophages were almost 20-fold more responsive than were *Aoah^−/−^* macrophages. It thus seemed likely that cell-associated LPS, if fully acylated, could maintain macrophage tolerance for long periods *in vivo*. To localize the cell-associated LPS, we injected FITC-labeled LPS i.p. to *Aoah^−/−^* and *Aoah^+/+^* mice and harvested their peritoneal macrophages 10 days later. Anti-FITC antibodies were used to detect cell-associated LPS. We found that the majority of the LPS was intracellular (**Fig. 2 A–C**). *Aoah^+/+^* macrophages contained more LPS per cell than did *Aoah^−/−^* macrophages, because there were more macrophages in *Aoah^−/−^* mouse peritoneum after i.p. LPS injection [25]. Anticipating that there would be differences in the intracellular localization of acylated and partially deacylated LPS, we then studied the macrophages using immunofluorescence microscopy. We found LPS co-localized with the lysosome marker, LAMP1 (**Fig. 2 D–H**), but not with markers for ER (Calnexin), cis and medial Golgi (Giantin), trans-Golgi (TGN46) or early endosomes (Rab5a) (not shown) [34]. There was no evident difference in the intracellular location of acylated LPS (in *Aoah^−/−^* cells) and partially deacylated LPS (in *Aoah^+/+^* cells). Thus, both *Aoah^+/+^* and *Aoah^−/−^* peritoneal macrophages contain LPS in endolysosomes for at least 10 days after i.p. injection; at this time *Aoah^−/−^* macrophages are tolerant and *Aoah^+/+^* macrophages are not, consistent with the tolerant state being determined by LPS acylation status rather than by differential LPS localization within the cells.

### The peritoneal environment maintains macrophage reprogramming

**Figure 3:**
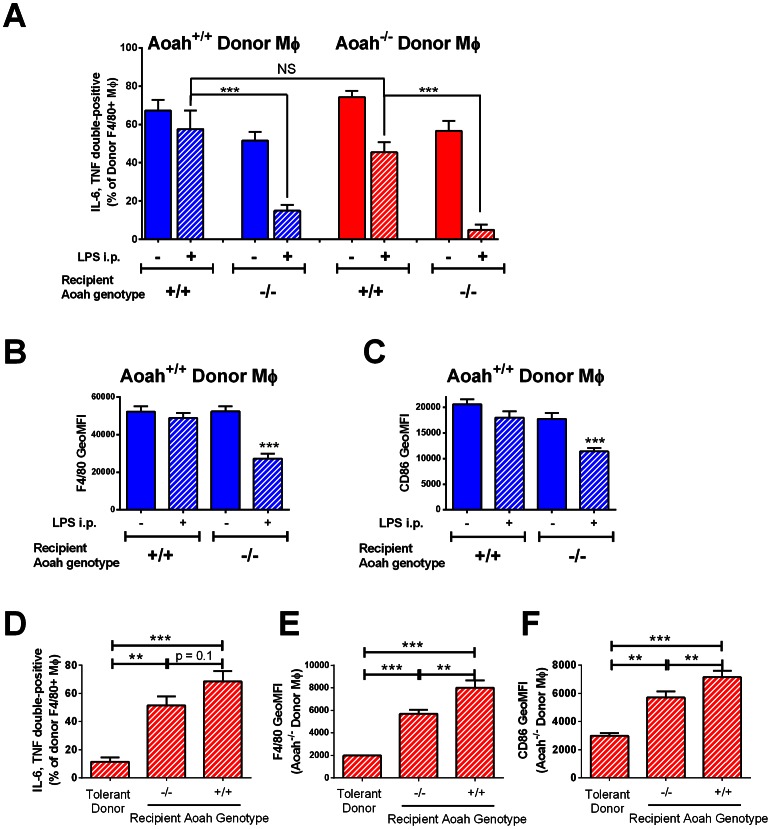
The peritoneal environment confers macrophage phenotype. (**A–C**) CD45.1 (or CD45.2) *Aoah^+/+^* or *Aoah^−/−^* macrophages were transferred to *Aoah^+/+^* or *Aoah^−/−^* recipient mice of the opposite CD45 genotype. 24 hours later, half of the mice in each group received 1 µg *E. coli* O14 LPS i.p. After 14 days, the peritoneal cells from the host mice were harvested. Half of the cells was treated with 1 µg/ml *E. coli* O111 LPS *ex vivo* for 4 hours in the presence of Brefeldin A. Intracellular IL-6 and TNF levels in F4/80+ donor macrophages (**A**) were measured using flow cytometry. Blue bars, *Aoah^+/+^* donor macrophages; red bars, *Aoah^−/−^* donor macrophages; diagonal markings indicate LPS exposure *in vivo*. The other half of the cells was used to measure F4/80+ macrophage surface expression of F4/80 (**B**) and CD86 without *ex vivo* stimulation (**C**). In B and C, only *Aoah^+/+^* donor macrophages were studied; they acquired the “tolerant” surface phenotype (low F4/80 and CD86 surface expression) when transferred to Aoah^−/−^ recipients. Tolerance tracked with the genotype of the host animal, not that of the donor macrophages. Data in A were combined from 4 experiments (n = 6–13/group); data in B and C were combined from 2 experiments, n = 6–8/group. (**D–F**) Tolerant *Aoah^−/−^* macrophages regain responsiveness in naïve mice. CD45.1 (or CD45.2) *Aoah^−/−^* mice were injected i.p. with 0.5 µg LPS. Fourteen days later, their peritoneal cells (including tolerant macrophages) were harvested and transferred i.p. to naïve *Aoah^−/−^* or *Aoah^+/+^* mice of the opposite CD45 type. After 7 days, peritoneal cells were harvested from the recipient mice and IL-6 and TNF responses were measured in the F4/80+ macrophages after re-challenging them with LPS *ex vivo* (**D**). F4/80 and CD86 surface expression was also measured (**E and F**). Only results from donor macrophages are shown. “Tolerant donor” macrophages were macrophages from *Aoah^−/−^* mice that received 0.5 µg LPS i.p. 21 days earlier and were freshly isolated (**in panel D**) or macrophages harvested from *Aoah^−/−^* mice that received 0.5 µg LPS i.p. 14 days earlier and were preserved in 10% DMSO, 90% FBS at −80°C until analysis (**in panels E and F**). Data were combined from 3 experiments. n = 6–13. **, P<0.01; ***, P<0.001. Tolerance was lost by 7 days after LPS-exposed macrophages were transferred to either naïve *Aoah^−/−^* or *Aoah^+/+^* mice.

We then tested whether prolonged tolerance *in vivo* is due to retention of bioactive LPS in *Aoah^−/−^* peritoneal macrophages or conferred by the peritoneal environment in which the macrophages reside. In these and subsequent transfer experiments, donor and recipient macrophages were identified by their surface expression of CD45.1 or CD45.2 using flow cytometry. We transferred *Aoah^+/+^* or *Aoah^−/−^* peritoneal cells to *Aoah^+/+^* or *Aoah^−/−^* recipient mice and injected LPS i.p. 24 hours later. Fourteen days after injection, we harvested peritoneal cells and stimulated them *ex vivo* with LPS while blocking protein secretion with Brefeldin A. We then used flow cytometry to identify F4/80+ macrophages, gated to distinguish CD45.1 cells from CD45.2 cells, and measured macrophage intracellular IL-6 and TNFα as indices of LPS responsiveness (see example in **Fig. S2**). We found that *Aoah^+/+^* macrophages were tolerant 14 days after they were transferred into *Aoah^−/−^* mice, whereas *Aoah^−/−^* macrophages exhibited reduced tolerance after they were transferred into *Aoah^+/+^* mice (**Fig. 3A**). *Aoah^+/+^* donor macrophages transferred into LPS-injected *Aoah^−/−^* mice also had lower surface levels of F4/80 and CD86 when studied 14 days after LPS injection, confirming that *Aoah^+/+^* macrophages gained the tolerant phenotype in LPS-injected *Aoah^−/−^* peritoneum (**Fig. 3B, C**). The findings were similar whether we transferred CD45.1 donor cells to CD45.2 recipient mice or vice versa. The presence of reprogramming 14 days after i.p. LPS injection was thus determined by the recipient environment, not by donor macrophage expression of AOAH or the lack thereof.

If macrophage tolerance is maintained for prolonged periods by environmental cues, removing tolerant *Aoah^−/−^* macrophages from an LPS-containing environment should allow them to regain responsiveness to LPS. We injected *Aoah^−/−^* mice i.p. with LPS to produce tolerant macrophages. Fourteen days after injection, the peritoneal cells were harvested, washed and transferred to the peritoneal cavity of a naïve *Aoah^+/+^* or *Aoah^−/−^* mouse. Seven days after transfer, we tested whether the donor *Aoah^−/−^* macrophages remained tolerant. We found that the donor *Aoah^−/−^* macrophages recovered from tolerance after they were transferred into either *Aoah^−/−^* or *Aoah^+/+^* mice (**Fig. 3D–F**); removal from an LPS-containing environment thus allowed macrophage recovery even in the absence of AOAH. The results again suggest that the *in vivo* environment plays a pivotal role in determining the behavior of peritoneal macrophages.

### LPS exposure can induce prolonged macrophage reprogramming

**Figure 4:**
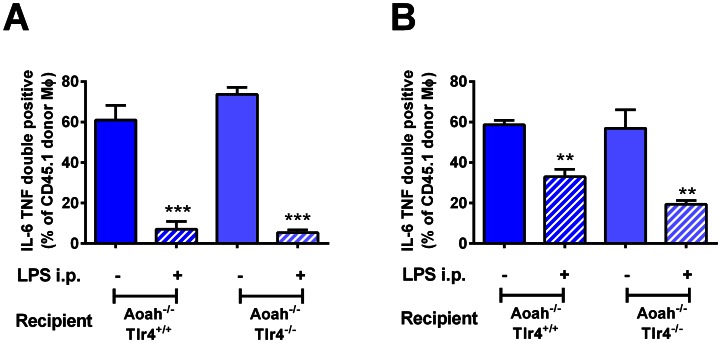
Bioactive LPS in the peritoneum is sufficient to maintain macrophage tolerance *in vivo*. (**A**) CD45.1 *Aoah^+/+^* peritoneal cells were transferred to CD45.2 *Aoah^−/−^Tlr4^+/+^* or *Aoah^−/−^Tlr4^−/−^* mice. Half of the mice in each group received 1 µg LPS i.p. Fourteen days later, the CD45.1 donor macrophages' IL-6 and TNF responses to LPS were determined following *ex vivo* re-challenge. Naïve *Aoah^+/+^* macrophages became tolerant when they were carried in LPS-injected *Aoah^−/−^* mice, whether or not the host mice expressed TLR4. n = 4–7. (**B**) CD45.2 *Aoah^−/−^Tlr4^+/+^* or *Aoah^−/−^Tlr4^−/−^* mice received 1 µg LPS i.p. as indicated. Fourteen days later, CD45.1 naïve *Aoah^+/+^* peritoneal cells were transferred i.p. to PBS- or LPS-injected mice. After 24 hours, the responses of the donor macrophages to LPS were measured *ex vivo*. Exposure to the LPS-containing *Aoah^−/−^* peritoneal environment rendered naïve *Aoah^+/+^* macrophages tolerant, whether or not the host mouse was able to respond to the i.p. dose of LPS. Data were combined from 2 experiments. n = 4–6. **, P<0.01; ***, P<0.001. Blue bars, *Aoah^−/−^ Tlr4^+/+^* recipients; light blue bars, *Aoah^−/−^Tlr4^−/−^* recipients; diagonal markings represent i.p. LPS injection.

We then asked how the peritoneal environment determines the fate of macrophages. Although bioactive LPS might still be present in the peritoneum, LPS also induces a broad array of inflammatory mediators, some of which (e.g., IL-10, TGF-β, IL-1 receptor antagonist) may promote macrophage reprogramming [15]. To find out whether wildtype macrophages can become tolerant in an *Aoah^−/−^* environment that lacks LPS-induced mediators, we transferred *Aoah^+/+^* CD45.1 peritoneal cells into mice that lack both AOAH and TLR4 (*Aoah^−/−^Tlr4^−/−^* CD45.2) and can neither deacylate, nor respond to, LPS. Fourteen days after i.p. LPS injection, the donor *Aoah^+/+^* CD45.1 macrophages were tolerant (**Fig. 4A**); since the recipient mice do not produce LPS-induced mediators, this observation suggested strongly that such mediators are not required to maintain prolonged tolerance *in vivo*. In another approach, CD45.2 *Aoah^−/−^Tlr4^+/+^* or *Aoah^−/−^Tlr4^−/−^* mice were given LPS i.p. Fourteen days later, CD45.1 naïve *Aoah^+/+^* peritoneal cells were introduced into the peritoneal cavity. One day after transfer, the donor macrophages had become tolerant in both *Aoah^−/−^Tlr4^+/+^* and *Aoah^−/−^Tlr4^−/−^* recipients (**Fig. 4B**). These findings suggested that bioactive LPS is present for a prolonged period in the LPS-exposed *Aoah^−/−^* peritoneum and that this LPS can induce and maintain macrophage tolerance in the absence of LPS-induced host mediators.

### Bioactive LPS can be recovered from LPS-injected *Aoah^−/−^* mice

**Figure 5:**
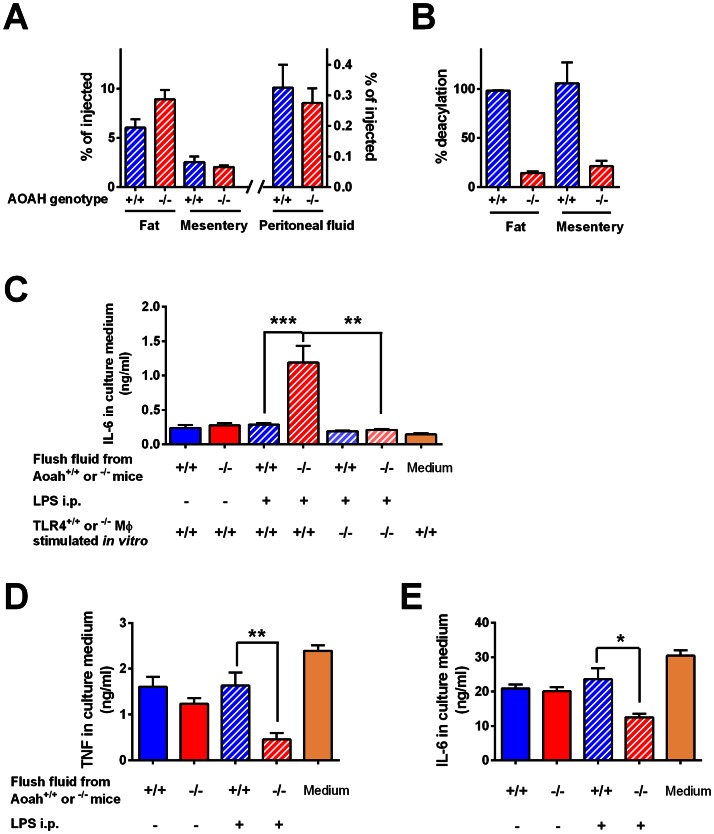
Bioactive LPS is present in the peritoneum 10 days after i.p. injection. (**A**) *Aoah^+/+^* or *Aoah^−/−^* mice were given 10 µg ^3^H/^14^C LPS i.p. Ten days later, their peritoneal cavities were flushed with 5 ml PBS that contained 5 mM EDTA and the peritoneal fat and mesentery were harvested. The amount of LPS in each specimen was determined by counting the ^14^C dpm in the LPS backbone. (**B**) The extent of deacylation was determined by measuring the ^3^H/^14^C ratio. Deacylation was not calculated in peritoneal fluid because the flush fluids contained low amounts of ^14^C and some free ^3^H- fatty acids. Blue striped bars, *Aoah^+/+^*; Red striped bars, *Aoah^−/−^*. (**C**) In other experiments, mice were injected i.p. with 10 µg LPS. Ten days later, the peritoneum was flushed with 2 ml cRPMI. The flush medium was centrifuged and the cell free supernatant was added to cultures of naïve *Tlr4^+/+^* or *Tlr4^−/−^* macrophages. Eighteen hours later, the culture medium was harvested to measure IL-6. Only peritoneal flush fluid from *Aoah^−/−^* mice elicited IL-6 production by naïve TLR4-expressing macrophages. (**D, E**) After incubation for 18 hours, the medium containing the flush fluid was removed, the macrophages were washed twice with cRPMI, and then they were treated with 1 µg/ml LPS for 6 hours. TNF and IL-6 in the culture medium were measured. Flush fluid from LPS-exposed *Aoah^−/−^* mice induced tolerance in naïve macrophages *in vitro*, whereas that from LPS-exposed *Aoah^+/+^* mice did not. Data are combined from 2 experiments. N = 6–8/group. *, P<0.05; **, P<0.01. Blue bars, *Aoah^+/+^* peritoneal flush medium overlying TLR4^+/+^ cells; red bars, *Aoah^−/−^* flush medium overlying TLR4^+/+^ cells; light blue bars, *Aoah^+/+^* flush medium overlying TLR4^−/−^ cells; pink bars, *Aoah^−/−^* flush medium overlying TLR4^−/−^ cells; diagonal markings indicate *in vivo* LPS exposure; orange bar, cRPMI medium overlying TLR4^+/+^ cells.

To obtain direct evidence for the presence of LPS in the peritoneum many days after i.p. injection, we injected 10 µg [^3^H/^14^C]LPS into the peritoneal cavities of *Aoah^−/−^* and *Aoah^+/+^* mice and measured ^14^C and ^3^H in the peritoneal flush medium, peritoneal cells, mesenteric membranes, and fat 10 days later. The distribution of ^14^C dpm, a marker for the LPS carbohydrate backbone, was similar in the presence and absence of AOAH: about 0.3% of the injected LPS was found in cell-free peritoneal flush fluid; from 6 to 9% of the injected ^14^C LPS was recovered from intraperitoneal fat and 2 to 3% from the mesentery (**Fig. 5A**). We reported previously that 1–4% of the injected LPS could be recovered from peritoneal cells [25]. Based on the ratio of ^3^H to ^14^C in the samples [35], 99% (*Aoah^+/+^*) and 19% (*Aoah^−/−^*) of the recovered LPS had been deacylated (**Fig. 5B**), in keeping with the results found for peritoneal cells [25].

Is the intraperitoneal acylated LPS bioactive? In other experiments, we gave 10 µg LPS i.p. to *Aoah^+/+^* and *Aoah^−/−^* mice and flushed their peritoneal cavities with culture medium 10 days later. The cell-free peritoneal flush medium from *Aoah^−/−^* mice activated naïve *Tlr4^+/+^* macrophages but not *Tlr4^−/−^* macrophages, suggesting that bioactive LPS was present in the medium (**Fig. 5C**). When we re-challenged the macrophages with LPS, we found that the flush medium from *Aoah^−/−^* mice could also render naïve *Tlr4^+/+^* macrophages tolerant (**Fig. 5D, E**). We conclude that (1) free LPS is present in the peritoneal fluid of *Aoah^−/−^* mice 10 days after i.p. injection; (2) this LPS is fully acylated; and (3) the acylated LPS is bioactive, able to activate naïve *Tlr4^+/+^* macrophages and reprogram them.

### Macrophages release bioactive LPS that can reprogram naïve macrophages

**Figure 6:**
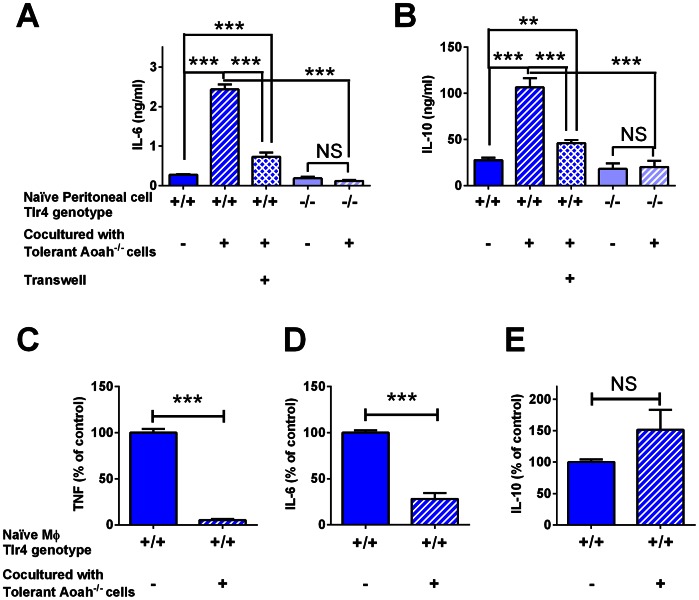
Co-culture with tolerant cells induces tolerance in naïve macrophages *in vitro*. (**A, B**) Peritoneal cells from naïve *Tlr4^+/+^* or *Tlr4^−/−^* mice were co-cultured for 18 hours at 37°C with washed peritoneal cells from *Aoah^−/−^* mice that had been injected with 10 µg LPS i.p. 10 days earlier. IL-6 and IL-10 were measured in culture medium. Only *Tlr4^+/+^* cells co-cultured with tolerant peritoneal cells released IL-6 and IL-10. Separation of naïve *Tlr4^+/+^* cells from LPS-exposed *Aoah^−/−^* cells in Transwell cultures significantly decreased cytokine production, suggesting that cell-cell contact is important for delivery of bioactive LPS. (**C–E**) In the same experiments, after incubation for 18 hours the *Tlr4^+/+^* cells that had been co-cultured with tolerant cells were washed with cRPMI twice and then treated with 1 µg/ml LPS for 6 hours before TNF, IL-6 and IL-10 were measured in the culture medium. Naïve macrophages co-cultured with tolerant cells became reprogrammed. Blue bars, *Tlr4^+/+^* naïve macrophages; light blue bars, *Tlr4^−/−^* naïve macrophages; diagonal markings indicate co-culture with tolerant cells; checked markings represent Transwell cultures. Data were combined from 3 experiments. n = 6–10. ***, P<0.001; NS, No significant difference.

If LPS that is taken up by macrophages can be released to act on other cells, fully acylated LPS released from *Aoah^−/−^* cells should be able to activate naïve cells and reprogram them. We first tested this idea *in vitro*. We injected *Aoah^−/−^* mice with LPS i.p. and harvested and washed their peritoneal cells 10 days later, when almost all of the LPS was associated with macrophages [25]. We co-cultured the peritoneal cells (including tolerant macrophages) with naïve *Tlr4^+/+^* or *Tlr4^−/−^* peritoneal cells (including naïve macrophages) for 18 hours. Significantly higher IL-6 and IL-10 (**Fig. 6A, B**) levels were found in the culture medium of *Tlr4^+/+^* peritoneal cells co-cultured with tolerant *Aoah^−/−^* cells than in the medium of *Tlr4^−/−^* cells co-cultured with tolerant cells, suggesting that LPS from tolerant cells stimulated naïve *Tlr4^+/+^* cells to produce these cytokines. After incubation for 18 hours, the co-cultured cells were washed twice with cRPMI and the adherent macrophages were treated with LPS for 6 hours (**Fig. 6C–E**). Naïve macrophages co-cultured with tolerant cells produced less TNF and IL-6, but similar amounts of IL-10, suggesting that they had become reprogrammed during the 18 hour incubation period.

When we co-cultured naïve *Tlr4^+/+^* peritoneal cells with *Tlr4^−/−^Aoah^−/−^* peritoneal cells that had been exposed to LPS *in vivo*, the naïve cells could also be activated, confirming that LPS from previously exposed macrophages is sufficient to activate naïve macrophages (data not shown). Co-culture of naïve *Tlr4^+/+^* peritoneal cells with LPS-exposed *Aoah^+/+^* cells (again, harvested 10 days after i.p. LPS injection, sufficient time for LPS inactivation to occur in AOAH-sufficient animals) did not induce IL-6 or IL-10 production (data not shown).

To find out whether direct cell-cell contact is required for naïve macrophage activation during co-culture, we separated naïve peritoneal cells from LPS-exposed peritoneal cells in transwell cultures. This significantly decreased IL-6 and IL-10 production by the naïve macrophages (**Fig. 6A, B**), suggesting that direct cell-cell contact enables optimal delivery of LPS from one macrophage to another.

**Figure 7:**
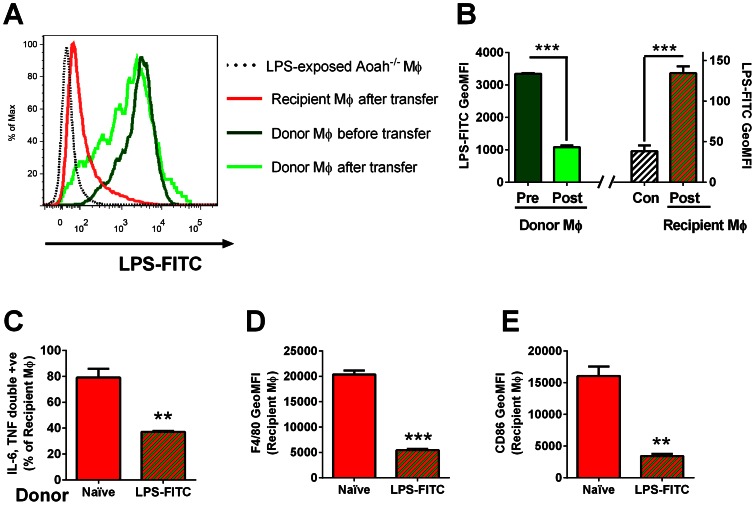
LPS can be released from macrophages and induce tolerance in other cells *in vivo*. (**A**) CD45.2 *Aoah^−/−^ Tlr4^−/−^* donor mice were injected i.p. with 20 µg LPS-FITC. Seven days later, their peritoneal cells were harvested, washed and transferred to CD45.1 *Aoah^−/−^* recipient mice. After 7 days, the donor and recipient F4/80+ macrophages were analyzed by flow cytometry to measure the amount of cell-associated LPS-FITC. Dark green, donor macrophages before transfer; light green, donor macrophages recovered 7 days after transfer; red, recipient peritoneal macrophages 7 days after transfer; Dotted black, LPS (unlabeled)-exposed *Aoah^−/−^* macrophages (autofluorescence control). (**B**) Geometric mean fluorescence intensity (GeoMFI) of cells in (**A**). (**C–E**) **Naïve recipient macrophages become tolerant after exposure** ***in vivo*** **to donor macrophages that contain LPS-FITC**. CD45.2 *Aoah^−/−^ Tlr4^−/−^* macrophages, either naïve or containing LPS-FITC, were transferred to naïve CD45.1 *Aoah^−/−^* mice as described in (**A**). Seven days after transfer, peritoneal cells were harvested from the recipient mice and half of the cells were re-challenged *ex vivo* with 1 µg/ml LPS in the presence of Brefeldin A. Intracellular IL-6 and TNF was measured in CD45.1 recipient macrophages (F4/80+) (**C**). Recipient macrophage surface F4/80 (**D**) and CD86 (**E**) were measured in cells that were not re-stimulated *ex vivo*. TLR4-deficient donor cells bearing LPS-FITC induced tolerance in recipient macrophages *in vivo*. Similar results were obtained in 2 additional experiments, each with n = 3. **, P<0.01; ***, P<0.001.

To test whether LPS-laden macrophages can release LPS to act on other macrophages *in vivo*, we injected 20 µg LPS-FITC i.p. to CD45.2 *Aoah^−/−^Tlr4^−/−^* mice. Seven days later, peritoneal cells were harvested, washed, and transferred i.p. to CD45.1 *Aoah^−/−^* naïve mice. After 7 days, we found that the transferred F4/80+ macrophages had lost 2/3 of their LPS-FITC and that recipient macrophages had gained small amounts of LPS-FITC, demonstrating that LPS can be released from donor macrophages and taken up by recipient macrophages *in vivo* (**Fig. 7A, B**). In addition, we found that the recipient macrophages became tolerant (**Fig. 7C–E**), indicating that they had been exposed to bioactive LPS (the *Tlr4^−/−^* donor cells should not produce tolerizing mediators).

These results show that macrophages can release the LPS that they contain and that the released LPS can act on other cells *in vivo*. If the LPS remains bioactive, it can induce tolerance in other macrophages.

### LPS-induced mediator(s) can induce tolerance in *Tlr4^−/−^* macrophages

**Figure 8:**
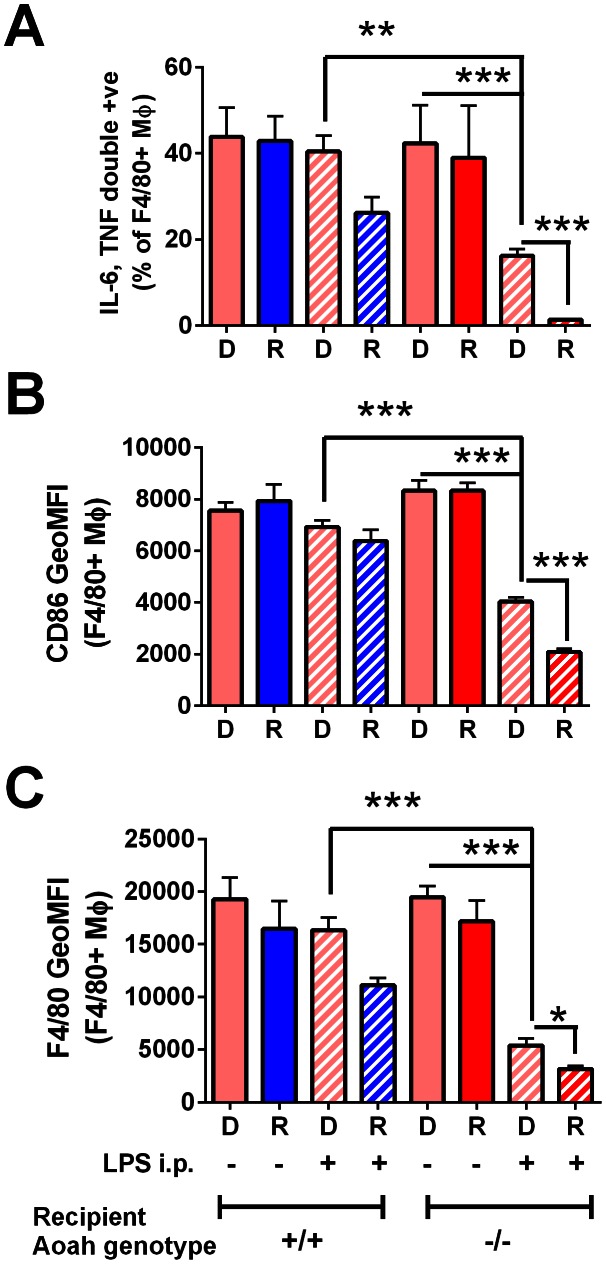
*Aoah^−/−^Tlr4^−/−^* macrophages can become tolerant in LPS-injected *Aoah^−/−^* mice. (**A**) CD45.2 *Aoah^−/−^Tlr4^−/−^* naïve peritoneal cells were transferred to the peritoneal cavities of CD45.1 *Aoah^+/+^Tlr4^+/+^* and *Aoah^−/−^Tlr4^+/+^* mice. After 18 hours, half of the recipient mice in each group were injected i.p. with 1 µg LPS. Fourteen days after injection, the peritoneal cells were harvested. Some of the cells were treated *ex vivo* with 40 µg/ml *Micrococcus luteus* plus 2.5 µg/ml poly I:C for 8 hours in the presence of Brefeldin A. IL-6 and TNF production by donor (D) and recipient (R) F4/80+ macrophages was measured using flow cytometry. (**B** and **C**) The remainder of the peritoneal cells was not re-stimulated *ex vivo* and was used to determine surface expression of F4/80 and CD86 on macrophages (F4/80+) by flow cytometry. **, P<0.01; ***, P<0.001. Data were combined from 2 independent experiments. n = 5–7. *Aoah^−/−^ Tlr4^−/−^* macrophages, which are unable to respond to LPS, became tolerant to TLR2 and TLR3 ligands (**A**) and developed the tolerant surface phenotype (**B** and **C**) when they were transferred into LPS-primed *Aoah^−/−^* mice, supporting a role for non-LPS stimuli in promoting tolerance *in vivo*. Pink bars, *Aoah^−/−^Tlr4^−/−^* donor macrophages; Blue bars, *Aoah^+/+^* recipient macrophages; red bars, *Aoah^−/−^* recipient macrophages; diagonal markings indicate *in vivo* LPS exposure.

Bioactive LPS is thus present in the peritoneum of LPS-injected *Aoah^−/−^* mice, where it is sufficient to prevent macrophages from regaining responsiveness. Do LPS-induced mediators also play a role in maintaining tolerance? We transferred CD45.2 *Aoah^−/−^Tlr4^−/−^* peritoneal cells to CD45.1 *Aoah^+/+^* or *Aoah^−/−^* (both *Tlr4^+/+^*) recipient mice, injected LPS i.p., and asked whether the *Aoah^−/−^Tlr4^−/^* ^−^ donor macrophages became tolerant in the peritoneum of tolerant *Aoah^−/−^* recipient mice. Fourteen days after LPS injection, we harvested recipient peritoneal cells and treated them *ex vivo* with *Micrococcus luteus* plus poly I:C (LPS-exposed *Aoah^−/−^* macrophages express hetero-tolerance [36] to *M. luteus* [TLR2 agonist] and poly I:C [TLR3 agonist] [25]). *Aoah^−/−^Tlr4^−/−^* donor macrophages (F4/80+) harvested from LPS-injected *Aoah^−/−^* recipients had significantly lower IL-6 and TNF responses to *ex vivo* re-challenge (**Fig. 8A**), and they also had less surface F4/80 and CD86 expression (**Fig. 8B, C**). Since *Aoah^−/−^Tlr4^−/−^* donor macrophages cannot sense LPS, these results suggested that LPS-induced mediators in the peritoneum are also important for prolonging tolerance in *Aoah^−/−^* mice. *Aoah^−/−^Tlr4^−/−^* donor cells were less tolerant than were the recipient's *Aoah^−/−^Tlr4^+/+^* macrophages, again reinforcing the prominent direct role played by fully acylated LPS.

### rhAOAH prevents prolonged tolerance *in vivo*

IFN-γ can improve monocyte function in septic patients and both IFN-γ and GM-CSF can restore responses of LPS-desensitized (tolerant) monocytes *in vitro* [37], [38]. We next tested whether *Aoah^−/−^* tolerant macrophages can recover their responses to LPS by treating them with rhAOAH (recombinant human AOAH), IFN-γ , GM-CSF or a combination of these agents for 18 hours *ex vivo* (**Fig. S3A–C**). rhAOAH only slightly increased the macrophages' responses, possibly because deacylation occurs slowly and would not be expected to reach completion within 18 hours [39]. GM-CSF preferentially restored the IL-6 response while IFN-γ mainly boosted the TNF and RANTES responses. Combining the three agents largely restored the macrophages' responses. *Aoah^−/−^* tolerant macrophages, like desensitized human monocytes [38], could thus be rescued by treating them with IFN-γ and GM-CSF *in vitro*.

**Figure 9:**
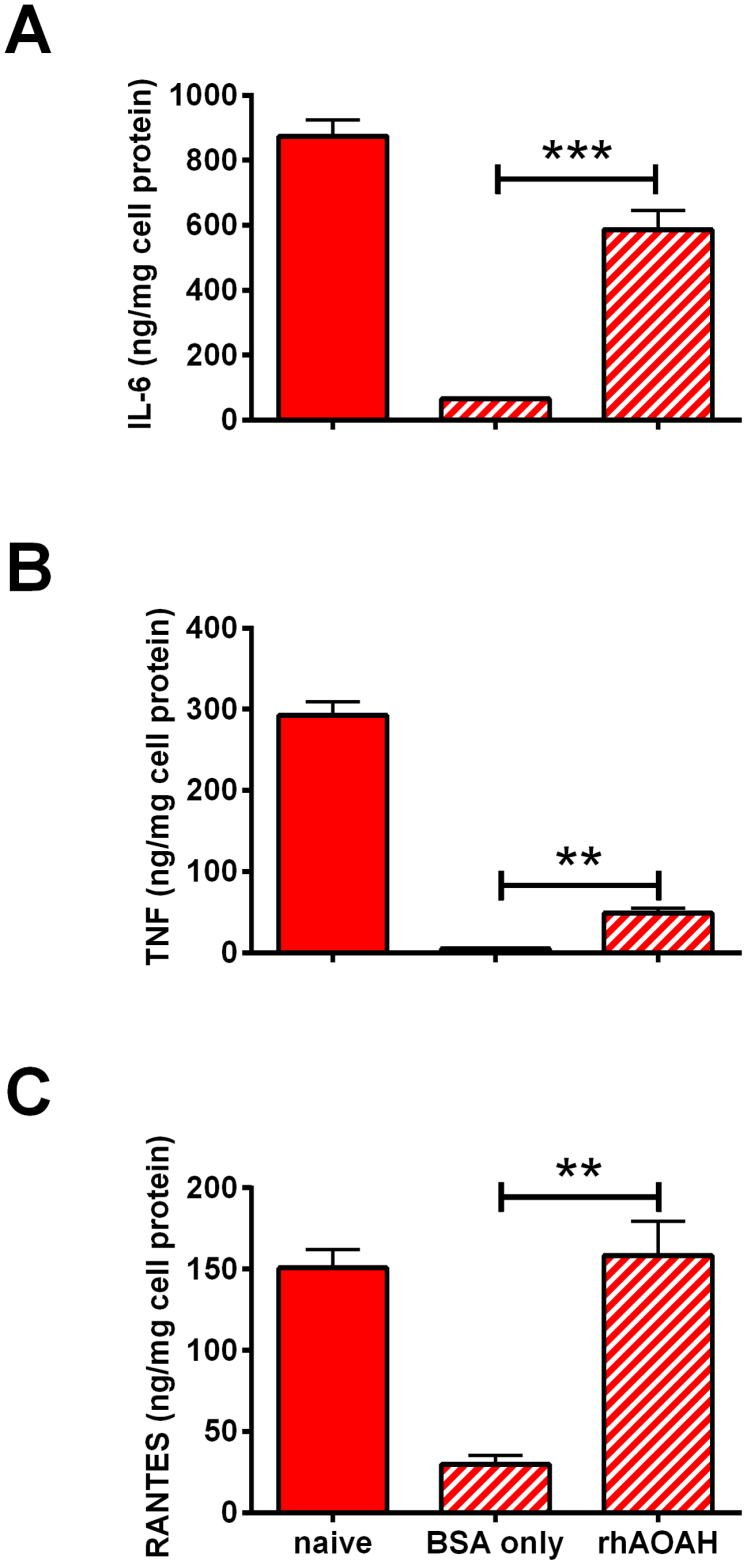
rhAOAH prevents prolonged endotoxin tolerance *in vivo*. *Aoah^−/−^* mice were injected with 1 µg LPS i.p. on day 0. From days 1 to 13, mice were given daily i.p. doses of 0.3 µg rhAOAH or placebo (carrier protein BSA in PBS) (diagonal marking red bars). Peritoneal cells were explanted on day 14 and the adherent macrophages were challenged *ex vivo* with LPS for 6 hrs before medium cytokine levels were measured. (**A**), IL-6; (**B**), TNF; (**C**), RANTES. Naïve Aoah^−/−^ peritoneal macrophages were used as controls (solid red bars). **, P<0.01; ***, P<0.001. rhAOAH largely prevented prolonged tolerance *in vivo*.

To test whether providing AOAH to mice can prevent or reverse prolonged tolerance *in vivo*, we gave *Aoah^−/−^* mice LPS i.p. on day 0, followed by rhAOAH or carrier protein BSA i.p. daily from day 1 to day 13. Peritoneal cells were harvested on day 14, plated, and adherent macrophages were rechallenged *ex vivo* with LPS. LPS-stimulated RANTES and IL-6 were at naïve Aoah^−/−^ macrophage levels in macrophages from animals that had received rhAOAH treatment (**Fig. 9A–C**). TNF production is the most sensitively reprogrammed component of tolerance in these cells; here the TNF level in culture medium overlying macrophages from LPS-exposed rAOAH-treated mice was significantly lower than the naïve macrophage level but 10-fold higher than the levels produced by macrophages from mice that received carrier protein BSA instead of rhAOAH. Recombinant AOAH was thus able to ameliorate prolonged tolerance *in vivo* in *Aoah^−/−^* animals. Treatment with IFN-γ or antibody to IL-10 receptor did not rescue *Aoah^−/−^* animals from prolonged endotoxin tolerance (data not shown), in line with the conclusion that bioactive LPS plays a dominant role in maintaining prolonged tolerance *in vivo*.

## Discussion

Patients who experience severe sepsis develop a state of immune tolerance that may last for many weeks and is thought to be immunosuppressive [40]. The phenomenon has been characterized principally by studying peripheral blood leukocytes, whose responses to LPS and other microbial agonists are typically altered to diminish pro-inflammatory cytokine production while maintaining or increasing production of anti-inflammatory molecules such as IL-10 and IL-1 receptor antagonist. LPS-induced tolerance in mice mimics the human phenomenon in many ways (reduced monocyte-macrophage CD86, reprogrammed cytokine production) but not in others (e.g., murine macrophages do not decrease class II molecule expression) [23].

Although some Gram-negative bacteria can modify the acylation structure of their LPS in ways that may alter its ability to trigger signaling via MD-2—TLR4 [41], LPS is also disabled by host enzymes, either on mucosal surfaces (alkaline phosphatase) [42] or in tissues (AOAH) [24]. In addition to experiencing prolonged tolerance, mice that lack AOAH respond to small subcutaneous or intravenous doses of LPS by producing high titers of polyclonal antibodies [43] and developing massive, prolonged hepatomegaly [44], [45]. Animals have many other mechanisms for neutralizing hexaacyl LPS in plasma and tissues [46], yet none of these is able to prevent these striking reactions in animals that cannot deacylate LPS. Transgenic mice that produce greater than normal amounts of AOAH are protected from live *E. coli* challenge [47], again emphasizing the importance of AOAH mediated LPS inactivation in optimizing anti-bacterial immune responses.

Continued exposure to microbial agonists can prolong the activation of cultured cells. Hume et al. reported that continuous exposure to LPS induced a sustained activation state in a macrophage cell line [48], and Hedl et al. found that prolonged exposure to muramyl dipeptide, a ligand for Nod2, promoted tolerance in human monocyte-derived macrophages [20]. Tolerance has also been described in human cells that may be exposed repeatedly to LPS *in vivo*, such as the alveolar macrophages of tobacco smokers [49] and blood monocytes from patients with uncontrolled gram negative bacterial infection [50] or cystic fibrosis [51]. Tolerance lasts for weeks in patients who have chronic pyelonephritis with active bacterial urinary tract infection [52], as well as in volunteers with typhoid fever [53]. Individuals who inhale endotoxin-rich agricultural dusts may also develop chronic macrophage activation or tolerance [54], [55]. Here we show that tolerance can be maintained *in vivo* for long periods by the presence of small amounts of bioactive LPS, raising the possibility that the rate and/or extent of LPS inactivation might influence the rate of recovery from many Gram-negative bacterial diseases.

The mammalian MD-2—TLR4 LPS receptor is activated most sensitively by LPSs that have a lipid A moiety that contains 6 acyl chains. Such “hexaacyl” LPSs are produced by many of the Gram-negative bacterial commensals and pathogens that can inhabit the mucosal surfaces of the upper respiratory and gastrointestinal tracts [56], [57]. Since AOAH inactivates these LPSs, AOAH deficiency might be associated with greater susceptibility to, or duration of, Gram-negative bacterial diseases that involve mucosal surfaces. To date, genetic linkage studies have found associations between polymorphisms in the AOAH gene and rhinosinusitis (confirmed in 2 populations of different ethnic composition [58], [59]) as well as asthma [60] in humans. In addition, two studies of large populations [61], [62] have independently found that AOAH mRNA expression is associated in *trans* with polymorphisms in HLA-DRB1 that, in turn, have been strongly linked to colitis and primary biliary cirrhosis. How AOAH influences disease expression, if it does, remains uncertain.

The present studies identified the presence of cell-associated and extracellular (cell-extrinsic) LPS as the primary determinant of prolonged LPS tolerance in macrophages *in vivo*. This conclusion is supported by the observations that 1) prolonged tolerance could be induced in either *Aoah^−/−^* or *Aoah^+/+^* macrophages that had been transferred into LPS-injected *Aoah^−/−^* mice, and 2) *Aoah^−/−^* macrophages did not exhibit prolonged tolerance when they were transplanted into *Aoah^+/+^* hosts, indicating that LPS inactivation by AOAH in the host environment, not in the tolerant cell itself, determines the tolerant phenotype. Tolerance could be induced in naïve macrophages either by direct contact with cells that had taken up LPS or by extracellular LPS acquired within the peritoneal environment. In addition, administration of rAOAH to *Aoah^−/−^* mice partially prevented tolerance, and tolerance could be induced in naïve macrophages when they were transferred into LPS-exposed *Aoah^−/−^Tlr4^−/−^* mice or co-cultured with LPS-exposed *Aoah^−/−^Tlr4^−/−^* peritoneal cells, which do not produce LPS-induced mediators but can release fully acylated LPS into their environment [26], [63], [64]. Although these studies identify bioactive LPS as essential for maintaining tolerance, we also found that LPS-induced paracrine mediators further promoted tolerance, perhaps in part by extending the phenotype to cells that do not express TLR4. These results also do not exclude the possibility that the epigenetic changes shown to be induced by short-term exposure to LPS [28]–[32] contribute to maintaining prolonged tolerance in macrophages, but these changes evidently do not persist (or maintain dominance) in an environment that lacks extracellular LPS; they can also be overcome by soluble mediators such as interferon-γ or GM-CSF.

Where does LPS deacylation occur *in vivo*? LPS may be deacylated extracellularly, as was shown using purulent ascites fluid [65]; both LPS-binding protein and CD14 can present LPS to extracellular AOAH in a manner that promotes its deacylation [66]. Alternatively, LPS may be taken up by macrophages, neutrophils, or dendritic cells and inactivated by AOAH, or conceivably LPS could be deacylated by AOAH in cells that have acquired AOAH via mannose-6-phosphate receptors [67]. Recent studies found that the LPS in circulating LPS-HDL (high density lipoprotein) complexes undergoes deacylation in the liver [68], where AOAH is produced by Kupffer and dendritic cells [44]. AOAH-deficient mice cannot deacylate LPS in ascites or in cells within, or on the walls of, the peritoneal cavity, and these cells or membranes may then become reservoirs that release small amounts of bioactive LPS over time. This LPS then could maintain macrophage tolerance or induce tolerance in monocytes that newly arrive in the peritoneal fluid. The LPS “depot” includes the peritoneal membrane and/or mesenteric fat, since we found significant quantities of radiolabeled, fully acylated LPS in these sites. Naïve macrophages may also acquire LPS from other macrophages by direct cell-cell contact.

Innate immune reactions to microbes are typically short-lived. Mobilizing an animal's antimicrobial armamentarium usually promotes microbial eradication and clearance within hours or a few days. Potentially harmful inflammation then resolves as the battlefield is cleared and defenses are restored [69]. Recovery is thought to involve both anti-inflammation (preventing inflammation-induced damage) and resolution (clearing the battlefield and promoting return of homeostasis). Known tissue resolution mechanisms include neutrophil apoptosis, macrophage emigration and efferocytosis of dead cells, and the production of lipoxins, resolvins [and other lipids] [70], proteases, and gaseous signals that promote restoration of homeostasis in tissues [69], [71], [72]. Here we present evidence for another essential component of resolution: inactivating the microbial molecules that tell the host that microbes are present. A host's ability to remove or disable bioactive microbial molecules from an infected tissue may influence the ultimate outcome of many host-microbe encounters, since inactivating these molecules removes an important obstacle to resolution of inflammation and restoration of innate host defenses.

## Materials and Methods

### Mice

*Aoah^−/−^* C57BL/6J mice were generated as described [35]. *Tlr4^−/−^* (B6.B10ScN-Tlr4lps-del/JthJ) mice were purchased from Jackson Laboratory. These mice have a 7 kb deletion in the TLR4 gene; the mutation was backcrossed to C57Bl/6J for at least 6 generations. C57BL/6J CD45.1 (B6.SJL-Ptprca Pepcb/BoyJ) mice were also from Jackson. *Aoah^−/−^ Tlr4^−/−^* and *Aoah^−/−^*CD45.1 mice were obtained by crossing *Aoah^−/−^* mice with *Tlr4^−/−^* or B6 CD45.1 mice, respectively. All mice were housed in a specific pathogen- and murine norovirus-free facility. All mice were studied using protocols approved by the Institutional Animal Care and Use Committee (IACUC). IACUC of the University of Texas Southwestern Medical Center (permit number: A3472-01) approved the Animal Protocol Number 0028-07-08-2. The IACUC of the National Institutes of Allergy and Infectious Diseases (permit number A4149-01) approved the Animal Study Protocol LCID 11E. Both protocols adhered to the Guide for the Care and Use of Laboratory Animals of the National Institutes of Health.

### Reagents

*E. coli* O14 LPS was prepared by the phenol-chloroform-petroleum ether method [73]. *E. coli* O111 LPS was purchased from Sigma. *Salmonella typhimurium* Rc LPS ([^3^H/^14^C]LPS; ^3^H-labeled fatty acyl chains and ^14^C-labeled glucosamine backbone) was prepared from *S. typhimurium* PR122 as described [74]; 1 µg had ∼150,000 dpm ^3^H and ∼10,000 dpm ^14^C. Experiments using radiolabeled LPS followed the requirements of the Radiation Safety department, UT-Southwestern Medical Center. FITC-LPS was prepared as described by Tobias et al. [25]. *Micrococcus luteus* cells and poly I:C were obtained from Sigma. Recombinant human AOAH was produced by ZymoGenetics, Inc. Recombinant mouse IFN-γ and recombinant mouse GM-CSF were from R&D Systems.

### Antibodies

Antibodies used for FACS were anti-F4/80 (clone BM8, eBioscience), anti-CD86 (clone GL1, BD), anti-FITC (polyclonal rabbit IgG, Invitrogen), anti-IL-6 (clone MP5-20F3, eBioscience), anti-TNF (clone MP6-XT22, eBioscience), anti-CD45.1 (clone A20, BD), and anti-CD45.2 (clone 104; BD). Antibodies used for microscopy were anti-CD107a (LAMP-1) (clone 1D4B, BD), anti-Rab5a (clone 15/Rab5, BD), anti-Giantin (rabbit polyclonal, Abcam), anti-TGN46 (rabbit polyclonal, Abcam), anti-Calnexin (rabbit polyclonal, Abcam).

### *Ex vivo* tolerance experiments

Mice were injected i.p. with various doses of LPS in 300 µl of PBS. Fourteen days later, the mice were euthanized using CO_2_ and their peritoneal cells were harvested by flushing the peritoneum with 5 ml of PBS containing 5 mM EDTA. Cells were stained with antibodies and analyzed using flow cytometry. To measure cell responses to re-challenge, peritoneal cells were washed and resuspended in cRPMI medium (RPMI 1640 containing 10% heat-inactivated FBS (endotoxin <0.06 EU/ml; Hyclone), 100 µM nonessential amino acids, 100 U/ml penicillin, 0.1 mg/ml streptomycin, 2 mM L-glutamine, 10 µM sodium pyruvate, 25 mM Hepes, pH 7.4, and 50 µM 2-mercaptoethanol) and plated with 1×10^6^ cells/well in 12-well-plates. After incubation for 18 hours (37°C, 5% CO_2_, 80% humidity), the floating cells were washed away and the adherent macrophages were treated with 1 µg/ml *E. coli* O111 LPS for 6 hours. The culture medium was used for ELISA and the cells were washed with PBS and lysed with PBS containing 0.1% Triton X-100 to measure protein (Biorad).

### Macrophage-associated LPS and imaging studies

Mice were injected i.p. with 10 µg LPS-FITC. Ten days after injection, peritoneal cells were harvested and stained with anti-F4/80 Ab to identify macrophages. The cells were then washed and fixed with 4% paraformaldehyde (PFA) or fixed and permeabilized with cytofix/cytoperm (BD). Anti-FITC PE antibody was used to detect cell-surface or total LPS-FITC by FACS. For microscopy, peritoneal cells were plated in culture dishes for 18 hours, then the floating cells were washed away and the adherent macrophages were fixed with 4% PFA before being blocked and permeabilized with 1% BSA, 25% goat serum, 0.05% saponin in PBS. Cells were stained with primary antibodies at 4°C overnight and with the secondary reagents at room temperature for 1 hour. 4′,6-diamidino-2-phenylindole (DAPI, Sigma) was used to stain nuclei. After washing, FluorSave aqueous mounting medium (EMD Chemicals) was applied and then coverslips were affixed. Stained sections were examined using a Leica SP5 X-WLL confocal microscope and analyzed using LAS AF Lite (Leica) software.

### Transfer experiments

Donor mice were euthanized and their peritoneal cells were harvested, washed, and resuspended in PBS. Cells from mice of the same genotype/treatment were pooled and an aliquot containing 2×10^6^ cells in 300 µl PBS was injected i.p to each recipient mouse. Because naïve Aoah^+/+^, Aoah^−/−^, Aoah^−/−^TLR4^−/−^ , LPS-injected Aoah^−/−^ mice and Aoah^−/−^TLR4^−/−^ mice have similar per cent distribution of macrophages [25], all recipient mice received approximately the same number of donor macrophages (8×10^5^). Twenty four hours after transfer, half of the recipient mice from each group received 1 µg LPS O14 i.p. Fourteen days later, recipient mice were euthanized and their peritoneal cells were harvested, washed, and resuspended in cRPMI medium containing 1 µg/ml LPS 0111 at 37°C for 4 hours (*Tlr4^+/+^* macrophages) or 40 µg/ml *Micrococcus luteus* plus 2.5 µg/ml poly I:C for 8 hours (*Tlr4^−/−^* macrophages) in the presence of 3 µg/ml Brefeldin A (eBioscience). The *ex vivo* stimulations were performed in non-tissue culture-treated V bottom 96-well plates (Sarstedt) to minimize macrophage adhesion. After stimulation, peritoneal cells were stained with anti-F4/80 antibody to identify macrophages and CD45.1 and CD45.2 antibodies to differentiate donor and recipient cells (see **Fig. S2**). The cells were then fixed and permeabilized (eBioscience) and stained with antibodies to IL-6 and TNF α to measure intracellular responses to *ex vivo* stimuli. We used the per cent of the total F4/80+ cells that were IL-6, TNF double positive as a measure of macrophage responsiveness. Approximately 1–10×10^4^ donor macrophages were recovered from each recipient mouse. Fewer donor macrophages were recovered from mice that had been injected with LPS, especially Aoah^−/−^ mice; this reflected the known migration of stimulated cells from the peritoneum [75]. In cases when few donor macrophages were present, at least 100 donor macrophages were measured by flow cytometry and results were compiled from at least 3 different mice in each group.

To exclude the possibility that macrophages became tolerant when cultured *ex vivo* with tolerant macrophages or vice versa, we harvested naïve (CD45.1) and tolerant (CD45.2) peritoneal cells, mixed them at different ratios and stimulated them with LPS for 4 hours *ex vivo*. The responsiveness of macrophages did not change during *ex vivo* co-culturing.

In some experiments (see **Fig. 4B**), we injected recipient mice i.p. with 1 µg LPS. Fourteen days later, naïve peritoneal cells (including naïve macrophages) were transferred i.p. to LPS-exposed mice or naïve mice. After 24 hours, peritoneal cells were harvested and the responsiveness of donor macrophages was analyzed *ex vivo* as described above. We also obtained tolerant macrophages from *Aoah* ^−/−^ mice that had been injected i.p with 0.5 µg LPS and transferred 2×10^6^ peritoneal cells (including approximately 8×10^5^ tolerant macrophages) to naïve *Aoah^−/^* ^−^ or *Aoah^+/+^* mice. Seven days later, we analyzed whether the tolerant macrophages had regained responsiveness in the naïve hosts *ex vivo* (Fig. 3D–E).

In other experiments (see **Fig. 7**), CD45.2 *Aoah^−/−^TLR4^−/−^* donor mice were injected with 20 µg LPS-FITC i.p. After 7 days, peritoneal cells from LPS-FITC injected mice or control naïve mice were harvested, washed, and 2×10^6^ peritoneal cells (including approximately 8×10^5^ macrophages) were transferred i.p. to CD45.1 *Aoah^−/−^* mice. Seven days after transfer, peritoneal cells were collected from recipient mice. The donor and recipient macrophages' LPS-FITC content, F4/80 and CD86 expression, and *ex vivo* IL-6 and TNF responses were measured by flow cytometry.

### ELISA assays

IL-6, TNF and IL-10 ELISA kits were purchased from BD, RANTES ELISA kit was from R&D system. Manufacturer instructions were followed.

### Quantitation of radiolabeled LPS

We injected 10 µg [^3^H/^14^C]LPS i.p. to *Aoah^−/−^* or *Aoah^+/+^* mice. Mice were euthanized 10 days later. Five ml PBS containing 5 mM EDTA was used to flush each peritoneal cavity. The peritoneal fat, mesentery and livers were harvested and homogenized in PBS. Aliquots were solubilized in 1 ml 0.5% SDS with 25 mM EDTA and 5 ml Bio-safe II scintillation cocktail (Research Products International Corp), and counted with quench and spill-over correction (Packard Tri-Carb 2100TR; Perkin-Elmer).

### Peritoneal flush medium and co-culture experiments

Mice were given 10 µg LPS i.p. Ten days later, they were euthanized using CO_2_ and 2 ml of cRPMI was used to flush the peritoneal cavity. The flush medium was centrifuged and the cell-free supernatant was collected. Peritoneal cells from naïve mice were cultured in cRPMI medium for 4 hrs to allow macrophages to adhere. The floating cells were washed away and the adherent macrophages were cultured in flush medium for 18 hours (37°C, 5% CO_2_, 80% humidity) before the medium was removed and saved for ELISA. The cells were then washed with cRPMI twice and the adherent macrophages were challenged with 1 µg/ml *E. coli* O111 LPS in cRPMI for 6 hours at 37°C. In co-culture experiments, 10^6^ tolerant cells were mixed with 10^6^ naïve cells in 12-well plates for 18 hours; the culture medium was collected for cytokine ELISA. The co-cultured cells were then washed with cRPMI twice and the adherent macrophages were stimulated with LPS for 6 hours. The culture medium was used for ELISA. To measure cytokines produced by naïve cells in the co-culture system and exclude a contribution from tolerant cells, 10^6^ tolerant cells were cultured in separate wells and treated in the same manner as were co-cultured cells. The low levels of cytokines produced by tolerant cells were subtracted from those produced by co-cultured cells.

To separate tolerant cells from naïve cells, 10^6^ tolerant cells were cultured in permeable Transwell inserts (Corning) overlying 10^6^ naïve cells in 12-well plates for 18 hours. The control was 10^6^ naïve cells in permeable inserts co-cultured with 10^6^ naïve cells. The culture media were collected after 18 hours of co-culture.

### *In vivo* rhAOAH treatment

*Aoah^−/−^* mice were given 1 µg LPS i.p. on day 0. From day 1 to day 13, mice were injected i.p daily with 0.3 µg rhAOAH or carrier protein BSA in 300 µl PBS. On day 14, the peritoneal cells were harvested and macrophages were challenged with 1 µg/ml *E. coli* O111 LPS for 6 hours at 37°C. Cytokine levels were measured in the culture medium.

### Flow cytometry

Flow cytometry analysis was done on FACS Calibur or LS Rortessa (BD). BD and FlowJo software was used to analyze data.

### Statistics

Unpaired Student's t test (two-tailed) was used for comparisons between groups. Linear regression was used to perform correlation analysis. In all figures, error bars indicate one SE.

## Supporting Information

Figure S1 **Macrophage TNF production after** ***ex vivo*** **LPS treatment correlates with cell SSC, F4/80 and CD86 expression.** *Aoah^+/+^* or *Aoah^−/−^* mice were injected i.p. with 0, 0.016, 0.08, 0.4, 2 or 10 µg *E. coli* O14 LPS. Each LPS dose was given to 3–5 *Aoah* ^+/+^ and *Aoah^−/−^* mice. Fourteen days later, their peritoneal cells were harvested. Some were used to measure macrophage (F4/80+) surface markers F4/80, CD86 and SSC by flow cytometry. Others were plated in culture dishes for 18 hours and the adherent macrophages were re-stimulated with *E. coli* O111 LPS (1 µg/ml) for 6 hrs. Medium TNF levels were measured by ELISA and correlated with SSC (**A**), F4/80 (**B**) and CD86 (**C**) surface expression on macrophages from the same mice. Each dot represents data from one mouse.
(TIF)

Figure S2 **Macrophage responses to** ***ex vivo*** **LPS stimulation.** In this example, CD45.1 peritoneal cells were transferred into peritoneum of CD45.2 recipient mice. Twenty-four hours later, recipient mice were injected with LPS i.p. Two weeks after injection, peritoneal cells were harvested from recipient mice and re-stimulated with 1 µg/ml LPS *ex vivo* for 4 hours in the presence of Brefeldin A. Cell surface F4/80, CD45.1, CD45.2 were stained first and then the cells were fixed and their intracellular IL-6 and TNF were stained. F4/80+ macrophages were gated (**A**); donor and recipient macrophages were differentiated by CD45.1 or CD45.2 expression (**B**); then donor and recipient macrophage intracellular IL-6 and TNF expression was plotted (**C**). We used the percentage of F4/80+ cells that was positive for both IL-6 and TNF to measure the macrophages' responses.
(TIF)

Figure S3 **Prolonged tolerance can be reversed** ***in vitro*** **.** Peritoneal cells were harvested from 1 µg LPS i.p. injected *Aoah^−/−^* mice. Macrophages were purified by letting them adhere to 12-well plates for 4 hrs and then they were treated with 400 ng/ml rhAOAH, 10 ng/ml IFNγ , 10 ng/ml GM-CSF or a combination of these agents *ex vivo* (red bars). Eighteen hours later, the macrophages were stimulated with LPS for 6 hours and cytokine levels were measured in culture media. (**A**), IL-6; (**B**), TNF; (**C**), RANTES. Peritoneal macrophages from 1 µg LPS-injected *Aoah^+/+^* mice were used as controls (blue bars). Tolerance reversal required a combination of the 3 agents.
(TIF)

Methods S1 **Microarray analysis.** IFN-γ, GM-CSF and rhAOAH treatment.
(DOCX)

Table S1 **Comparison of surface markers and mRNA expression in peritoneal macrophages from LPS-injected** ***Aoah^+/+^*** **and** ***Aoah^−/−^*** **mice.**
(DOCX)
