# Supplementary figures and images for: Persistently Active Microbial Molecules Prolong Innate Immune Tolerance In Vivo
Source: PLoS Pathog. 2013 May 9;9(5):e1003339. doi: 10.1371/journal.ppat.1003339 (PMC3649966; doi:10.1371/journal.ppat.1003339)

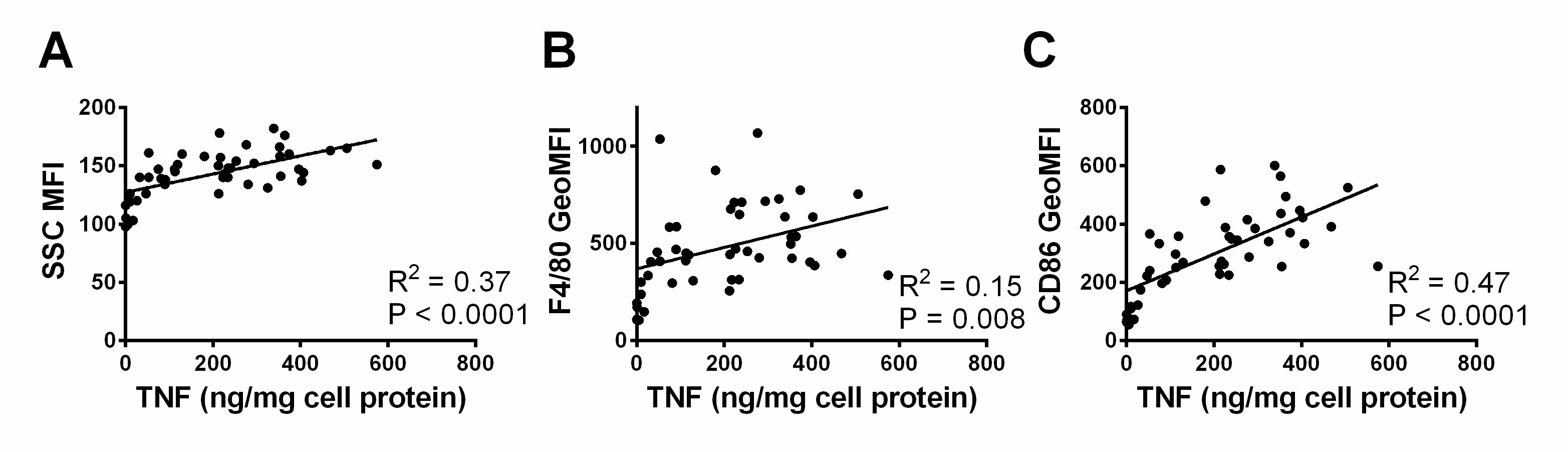

Supplement: Figure S1 — Macrophage TNF production after ex vivo LPS treatment correlates with cell SSC, F4/80 and CD86 expression. Aoah+/+ or Aoah−/− mice were injected i.p. with 0, 0.016, 0.08, 0.4, 2 or 10 µg E. coli O14 LPS. Each LPS dose was given to 3–5 Aoah +/+ and Aoah−/− mice. Fourteen days later, their peritoneal cells were harvested. Some were used to measure macrophage (F4/80+) surface markers F4/80, CD86 and SSC by flow cytometry. Others were plated in culture dishes for 18 hours and the adherent macrophages were re-stimulated with E. coli O111 LPS (1 µg/ml) for 6 hrs. Medium TNF levels were measured by ELISA and correlated with SSC (A), F4/80 (B) and CD86 (C) surface expression on macrophages from the same mice. Each dot represents data from one mouse. (TIF) [file ppat.1003339.s001.tif]

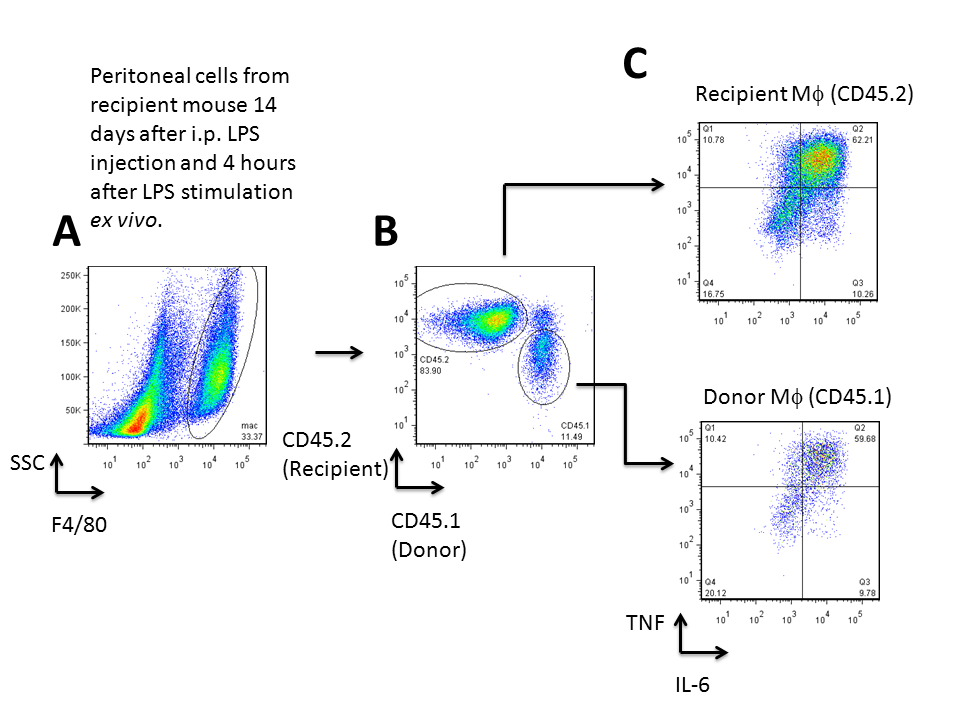

Supplement: Figure S2 — Macrophage responses to ex vivo LPS stimulation. In this example, CD45.1 peritoneal cells were transferred into peritoneum of CD45.2 recipient mice. Twenty-four hours later, recipient mice were injected with LPS i.p. Two weeks after injection, peritoneal cells were harvested from recipient mice and re-stimulated with 1 µg/ml LPS ex vivo for 4 hours in the presence of Brefeldin A. Cell surface F4/80, CD45.1, CD45.2 were stained first and then the cells were fixed and their intracellular IL-6 and TNF were stained. F4/80+ macrophages were gated (A); donor and recipient macrophages were differentiated by CD45.1 or CD45.2 expression (B); then donor and recipient macrophage intracellular IL-6 and TNF expression was plotted (C). We used the percentage of F4/80+ cells that was positive for both IL-6 and TNF to measure the macrophages' responses. (TIF) [file ppat.1003339.s002.tif]
