# Supplementary material for: Persistently Active Microbial Molecules Prolong Innate Immune Tolerance In Vivo
Source: PLoS Pathog. 2013 May 9;9(5):e1003339. doi: 10.1371/journal.ppat.1003339 (PMC3649966; doi:10.1371/journal.ppat.1003339)
Supplement: Methods S1 — Microarray analysis. IFN-γ, GM-CSF and rhAOAH treatment. (DOCX) [file ppat.1003339.s004.docx]

**Supporting Methods**

**Microarray analysis.**  Groups of 9 C57BL/6 *Aoah^+/+^* or *Aoah^-/-^* mice were given i.p. injections of 10 µg E. coli LPS/mouse. Twenty-one days later (when *Aoah^-/-^* mice remain tolerant and *Aoah^+/+^* mice have recovered), peritoneal macrophages were harvested and the yields from three mice were pooled to form 3 samples per group (i.e., three samples of *Aoah^+/+^* and *Aoah^-/-^* mice, with each sample comprised of peritoneal macrophages from 3 mice). Samples were then plated in 6 well plates and incubated overnight at 37°C, 5% CO2. The next day, non-adherent cells were washed off and RNA was isolated using RNeasy (Qiagen) per manufacturer’s instructions. The experiment was repeated, with similar results, using samples from *Aoah^+/+^* or *Aoah^-/-^* C3H/HeN mice. In addition, microarray analysis was performed on peritoneal macrophages from untreated *Aoah^+/+^* and *Aoah^-/-^* mice to assure that their unstimulated mRNA expression was concordant.

Microarray analysis was performed by the UT-Southwestern Microarray Core. Briefly, RNA quality and quantity was determined using the Agilent Bioanalyzer chip (RNA 6000 Nano kit). cDNA was then prepared for each group, labeled, and used to probe Illumina MouseWG-6_v1_1 arrays.

Data were analyzed using Illumina’s GenomeStudio v2010.1 software. Differential gene expression was analyzed using the *Aoah^+/+^* group as the principal group. Average signal intensities were normalized by cubic spline. Group normalization was performed using the Illumina Custom Model (built into GenomeStudio). Statistical significance was determined using GenomeStudio’s ‘Difference Score’ (DS), which combines detection p-value, average signal strength (after normalization), and difference p-value into a single statistical parameter. To estimate significant differences, we used a ‘Difference score’ of ≥ 30 or ≤ -30. This is a robust criterion corresponding to a difference p-value of ≤ 0.001.

Microarray data have been deposited in GEO (Gene Expression Omnibus) database supported by NCBI.

Platform #: GPL15943

Series #: GSE40154

Accession#s (samples):

     GSM986464; W-L-P (wildtype primed with LPS 21 days prior to harvest; PBS *ex vivo*)

     GSM986465; K-L-P (*Aoah ^-/-^* primed with LPS 21 days prior to harvest; PBS *ex vivo*)

     GSM986466; W-L-L (wildtype primed with LPS 21 days prior to harvest; LPS *ex vivo*)

     GSM986467; K-L-L (*Aoah ^-/-^* primed with LPS 21 days prior to harvest; LPS *ex vivo*)

**IFN-γ, GM-CSF and rhAOAH treatment**. *Aoah^-/-^* mice were injected with 1 μg LPS 014. Fourteen days later, their tolerant peritoneal macrophages were harvested, cultured in 12-well plates at 1 x 10^6^ cells/well and treated with 400 ng/ml rhAOAH, 10 ng/ml IFN-γ , 10 ng/ml GM-CSF or combination *ex vivo*. Eighteen hours later, 1 μg/ml LPS0111 was added for 6 hours and cytokine production was measured in the culture medium by using ELISA.
