# Supplementary material for: Persistently Active Microbial Molecules Prolong Innate Immune Tolerance In Vivo
Source: PLoS Pathog. 2013 May 9;9(5):e1003339. doi: 10.1371/journal.ppat.1003339 (PMC3649966; doi:10.1371/journal.ppat.1003339)
Supplement: Table S1 — Comparison of surface markers and mRNA expression in peritoneal macrophages from LPS-injected Aoah+/+ and Aoah−/− mice. (DOCX) [file ppat.1003339.s005.docx]

**Table S1**

**Comparison of surface markers and mRNA expression in peritoneal macrophages from LPS-primed *Aoah^+/+^* and *Aoah^-/-^* mice.**

|  | *Aoah^+/+^* | | *Aoah^-/-^* | | *Aoah^+/+^* | *Aoah^-/-^* |
| --- | --- | --- | --- | --- | --- | --- |
| Marker genes/proteins | PBS-primed | LPS-primed | PBS-primed | LPS-primed | LPS-primed | LPS-primed |
|  | Mean fluorescence intensity (MFI) | | | | Normalized signal (Microarray) | |
| SSC | 131 ± 7  N = 7 | 111 ± 11  N = 8 | 129 ± 5  N = 6 | 87 ± 5  N = 6  *** |  |  |
| F4/80 | 416 ±97  N = 7 | 318 ± 34  N = 8 | 419 ± 70  N = 6 | 124± 35  N = 6  *** | 6791 | 8976  ** |
| CD86 | 325 ± 38  N = 7 | 280 ± 46  N = 8 | 240 ± 30.21  N = 6 | 69 ± 8.5  N = 6  *** | 4336 | 1218  ** |
| CD11b | 1977 ± 358  N = 7 | 1850 ± 505  N = 8 | 2230 ± 224  N = 6 | 1582 ± 493  N = 6  * | 692 | 814  ns |
| CD69 | 21 ± 3.0  N = 3 | 10 ± 2.3  N = 4 | 17 ± 1.8  N = 3 | 13 ± 1.1  N = 3  * | 25 | 61 |
| CD16/32 | 97 ± 15  N = 3 | 96 ± 2  N = 3 | 97 ± 10  N = 3 | 63 ± 0  N = 3  ** | 27993 | 14790  ** |
| CD64 | 398 ± 13  N = 3 | 402 ± 13  N = 3 | 418 ± 19  N = 3 | 269 ± 11  N = 3  *** | 133 | 145 |
| Gr1 | 25 ± 4  N = 7 | 20 ± 1  N = 8 | 20 ± 0.9  N = 6 | 15 ± 1.6  N = 6  *** | 83 | 28 |
| CD40 | 80 ± 12.5  N = 3 | 80 ± 5.6  N = 4 | 64 ± 3.63  N = 3 | 100 ± 14.7  N = 3  * | 293 | 426  * |
| MHC-II | 42 ± 3.6  N = 3 | 32 ± 6.0  N = 3 | 37 ± 7.5  N = 3 | 34 ± 5.7  N = 3  NS | 12503 | 13808 |
| CD80 | 406 ± 28  N = 3 | 440 ± 58  N = 4 | 362 ± 29  N = 3 | 471 ± 29  N = 3  NS | 601 | 697 |
| Arginase | 53 ± 7  N = 3 | 45 ± 3  N = 3 | 32 ± 4  N = 3 | 36 ± 1  N = 3  NS | 3384 (Arg1)  995 (Arg2) | 776 ***  79 |
| FIZZ 1 | 105 ± 14  N = 3 | 107 ± 22  N = 3 | 107 ± 3  N = 3 | 58 ± 2  N = 3  *** | 4712 | 110 |

*, p < 0.05 (LPS-primed AOAH vs. other comparison group(s)); **, p < 0.01; ***, p < 0.001
